# Trends in Corticosteroid Injections for Treatment of Lateral Epicondylitis: An Analysis of 80,169 Patients

**DOI:** 10.5435/JAAOSGlobal-D-21-00186

**Published:** 2021-09-10

**Authors:** John Q. Sun, Quinn A. Stillson, Jason A. Strelzow, Lewis L. Shi

**Affiliations:** From the Pritzker School of Medicine, University of Chicago, Chicago, IL (Sun and Stillson), and the Department of Orthopaedic Surgery and Rehabilitation Medicine, University of Chicago Medicine and Biological Sciences, Chicago, IL (Dr. Strelzow and Dr. Shi).

## Abstract

Corticosteroid (CS) injections are a frequently used treatment modality for lateral epicondylitis (LE) despite an increasing number of studies suggesting their lack of efficacy. The objective of this study was to review the annual utilization of CS injections for treatment of LE, as well as that of other nonsurgical treatments and surgical treatments, to understand how recent publications have affected the practice of physicians in treating LE.

**Methods:**

Patients with LE from 2010 to 2017 were identified within a national insurance database and grouped by treatment modalities of CS injections, physical therapy, bracing treatment, and surgery. Epidemiologic and demographic data were reported using descriptive statistics. The number of patients receiving each treatment and the number of CS injections per patient were quantified for each year, and annual trends were analyzed using logistic regression.

**Results:**

Among 80,169 qualifying patients, 16,476 received CS injections, 12,180 received physical therapy, 1,874 received bracing treatment, and 2,650 underwent surgery, with patients receiving multiple modalities being members of each respective group. We found a significant decrease in the proportion of patients with LE receiving CS injections from 23.3% in 2010 to 18.8% in 2017 (R^2^ = 0.956, *P* < 0.001). Interestingly, the number of CS injections per patient increased during this period from 1.33 to 1.83 (R^2^ = 0.843, *P* = 0.001). No notable changes in utilization trends for other modalities were found.

**Discussion:**

Overall, our data support a decline in the use of CS injection as a treatment modality for LE from 2010 to 2017. Although correlational, this trend may reflect the increasing body of published evidence demonstrating the ineffectiveness of CS injections for the treatment of LE. In addition, the increasing number of injections per patient among those who received injections contrasts with the overall decrease in steroid utilization among all patients. Further study is needed to fully understand the mechanisms behind these trends.

Lateral epicondylitis (LE), commonly known as “tennis elbow,” affects 1% to 3% of the population annually and is associated with risk factors such as smoking, obesity, increased age, repetitive movements, and movements with high physical load.^1–3^ Overuse of the extensor muscles results in degenerative tendinopathy, most commonly that of the extensor carpi radialis brevis. This can result in tenderness of the lateral epicondyle and proximal wrist extensor muscle mass, painful resisted extension of the wrist with a flexed elbow, or painful passive flexion of the wrist with an extended elbow—impairments which can markedly hinder work-related and daily living activities.^4,5,6,7^ Poorer prognoses are associated with severe pain at presentation, concomitant neck pain, greater daily physical demands, and higher levels of baseline pain because such risk factors are associated with a longer duration of symptoms and greater pain scores 1 year from disease onset.^8,9^ Treatment of LE entails nonsurgical treatment such as activity modification, bracing treatment, NSAIDs, corticosteroid (CS) injections, and physical therapy (PT); surgery is rarely needed because most patients have symptom resolution within 1 year.^10,11,12,13,14,15,16^

There remains a lack of consensus regarding the preferred method of nonsurgical treatment of this condition.^17^ Among the most common of these nonsurgical practices is CS injections.^14^ Early studies demonstrated the effectiveness of CS in reducing LE pain 6 weeks after injection.^12,18,19,20,21^ However, more recent studies report an increased rate of relapse of LE pain and functional impairment, as indicated by measures of grip strength and pain rating, in patients who received CS injections compared with those who did not, with no notable benefit compared with controls at 3 months after treatment.^19,20,22^ Additional findings suggest that although CS can relieve acute LE symptoms, they are contraindicated for long-term cases, as evidenced by decreased rates of symptom improvement or resolution among patients receiving CS compared with those receiving other nonsurgical treatments.^14,22,23,24,25,26,27^ Additional concerns around increased rates of postoperative infection, need for revision surgeries, and the suggestion that CS injections may be less cost-effective than other nonsurgical treatments have been documented.^28–30^

Despite the growing contemporary evidence against CS injection use for treatment of LE, beginning with an impactful meta-analysis in 2010, current recommendations for CS injections in treating LE are to use them for short-term relief.^27,30,31^ Amidst these guidelines and the growing body of literature against the use of CS, it remains unknown if physician behavior has changed in treating LE. In this study, we examined the annual usage of CS injections for treatment of LE from 2010 through 2017, with the primary goal of determining if utilization of this treatment modality changed throughout the 8-year period. As a secondary goal, we will provide an overview on trends in diagnosis and treatment throughout this period by examining the annual utilization of other treatment modalities, including bracing treatment, PT, and surgery.

## Methods

The PearlDiver Patient Record Database (PearlDiver; www.pearldiverinc.com) is a publicly available national database of insurance billing records, which can be used to identify patients with an orthopaedic International Classification of Diseases, Ninth Revision (ICD-9) and 10th Revision (ICD-10) or Current Procedural Terminology (CPT) code assigned to procedures. This database was chosen because it includes a patient volume of over 121 million patients of all age groups and payer types, including commercial insurance, Medicare, Medicaid, and self-pay, from 2010 through 2018 (Q2).

**Figure 1:**
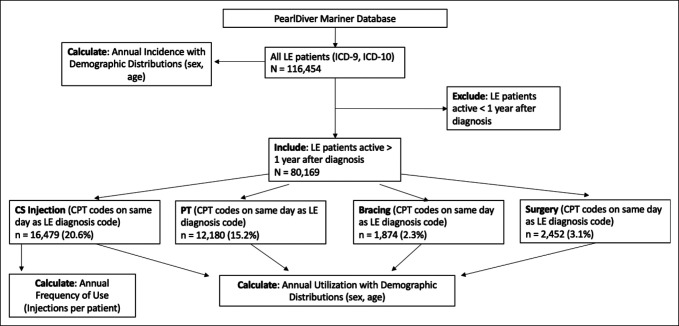
Flowchart showing study design. Patients with lateral epicondylitis (LE) identified in the PearlDiver database were stratified based on treatment modality to calculate annual proportions of patients with LE receiving each modality. Patients active <1 year were excluded from calculations of annual utilization and were only included in calculations of annual incidence.

The PearlDiver Mariner insurance database was used to identify all patients with LE from 2010 to 2017 using ICD-9 and ICD-10 codes (Appendix, http://links.lww.com/JG9/A152). To ensure patients were continuously represented in the database, we excluded patients who were active in the database for less than 1 year after diagnosis of LE (Figure 1). Within this group of patients with LE, we then subgrouped patients who had received CS injections for treatment of LE using CPT codes for therapeutic injections and accompanying CPT codes for CS joint injections (Appendix, http://links.lww.com/JG9/A152). To ensure accuracy of coding, only CS injection codes with concurrent ICD-9 or ICD-10 diagnosis codes for LE were included. Patients with LE receiving treatment modalities of PT, bracing treatment, and surgery were identified in the same manner—that is, all patients with LE were subgrouped according to treatment modality.

Epidemiologic and demographic data were reported using descriptive statistics. Incidence of LE was calculated per 10,000 patients using ICD-9 and ICD-10 codes and the total number of patients in the PearlDiver Mariner database for that given year.

Next, we quantified the total number of patients receiving each treatment modality by summing all of the most recent dates for each corresponding CPT code. Patients receiving each treatment modality in a given year were calculated by quantifying the number of final dates for each CPT code in that year from 2010 to 2017.

Within the subgroup of patients with LE receiving CS injections, we calculated the number of CS injections received per patient per year. We first stratified patients within this subgroup by their date of most recent CS injection, thereby quantifying the number of patients who received their most recent injection in each year from 2010 to 2017. Next, we calculated the total number of injections given to this subgroup for that given year. For each year, we divided the total number of injections given by the number of patients receiving their most recent injection, giving us the number of CS injections per patient for each year from 2010 to 2017.

Trends over time were analyzed using logistic regression. Significance was set for all analyses to a *P* value less than 0.05.

## Results

The initial query of the PearlDiver Mariner database yielded 116,454 patients with LE from 2010 to 2017. In addition, among the 116,454 total patients with LE, incidence of LE between 2010 and 2017 decreased from 16.25 to 11.56 new cases per 10,000 patients (*P* < 0.001). Among age groups, there was a significant decrease in new cases among patients aged 40 to 64 years and a significant increase in new cases among patients older than 64 years (*P* < 0.01). No significant changes were observed in the proportion of male and female new cases (see SDC Table 2, http://links.lww.com/JG9/A152).

**Figure 2:**
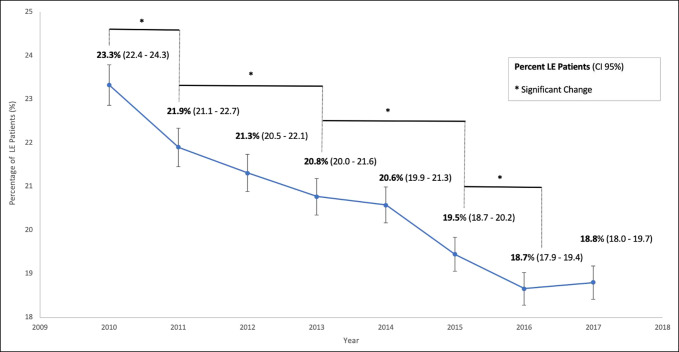
Graph showing trends in corticosteroid injection utilization for lateral epicondylitis (LE). Analysis of changes of annual proportions of patients with LE receiving CS injections from 2010 to 2017 using logistic regression. Significance was determined at *P* < 0.05.

Of the 116,454 patients with a diagnosis of LE, 80,169 were active in the database for at least 1 year from 2010 through 2017. Within this cohort of active patients, 16,476 received stable CS injections to treat LE (20.6%), 12,180 received PT (15.2%), 1,874 received bracing treatment (2.3%), and 2,452 received surgery (3.1%) (Table 1). Patients receiving multiple modalities were included in each respective group such that a patient receiving both therapy and CS injections was included in groups of the 12,180 and 16,476 patients, respectively. In addition, patients with LE receiving none of these treatment modalities were not included in any treatment subgroups. Furthermore, among the 80,169 active patients, analysis of CS utilization showed a significant decrease in the percentage of diagnosed patients receiving this treatment modality between 2010 and 2017 from 23.3% to 18.8% (*P* < 0.001). Analysis revealed no significant changes in proportions of usage of CS injections based on age and sex (see SDC Table 3, http://links.lww.com/JG9/A152). Significant decreases in overall utilization occurred every 1 to 2 years throughout this period (*P* < 0.05) (Figure 2). The average number of CS injections received per patient with LE increased from 2010 through 2017 from 1.33 to 1.83 injections per patient (*P* = 0.0013) (see SDC Table 3, http://links.lww.com/JG9/A152).

**Table 1:** Treatment Modalities for Lateral Epicondylitis

Modality	N (%)
Corticosteroid injection	16,479 (20.6)
Physical therapy	12,180 (15.2)
Bracing treatment	1,874 (2.3)
Surgery	2452 (3.1)
Total patients	80,169

Finally, analysis of associated treatment modalities including PT, bracing treatment, and the use of surgery demonstrated no notable change in their utilization for LE among the 80,169 active patients between 2010 and 2017. We did, however, find a significant change in the proportion of patients receiving PT among those aged 40 to 64 years and older than 64 years (see SDC Tables 4–6, http://links.lww.com/JG9/A152).

## Discussion

The purpose of this study was to report current trends in CS usage for the treatment of LE. We found a notable decrease in the utilization of this treatment modality, from 23.3% in 2010 to 18.8% of patients in 2017, with notable decreases occurring every 1 to 2 years throughout this period. In addition, our study showed a notable increase in the mean number of injections received per patient from 1.33 in 2010 to 1.83 in 2017.

Overall, these findings demonstrate a change in physician practice for the nonsurgical treatment of LE. This is especially evident given the lack of similar declines in alternative treatment modalities such as bracing treatment and PT and suggests this is a specific trend. We suggest this trend may reflect the increasing quantity of impactful studies indicating the lack of efficacy of this treatment modality for long-term cases. In addition to the studies by Coombes et al, other investigations have found evidence of CS injections having similar detrimental effects on tendinopathies of the rotator cuff, Achilles, and patellar tendon.^24^ These findings of increased incidences of symptom recurrence and pain exacerbation were first reported for LE as early as 1990.^32,33^

However, these earlier findings likely failed to change physician practice in treatment of tennis elbow at their time of publication because of small sample sizes and the lack of contemporary publications on the same topics.^31^ Reported CS utilization for tennis elbow varied heavily in the early 1990s between 14% and 38%.^18^ This variation persists in more recent studies, with 17% to 40% of tennis elbow patients receiving injections as recently as 2016.^34^

Although our study shows a decrease in overall utilization of CS in our population, this change is unlikely to be ubiquitous among providers, as demonstrated by observed increase in the number of injections received per patient throughout the same 8-year period. Previous database studies have shown a relative increase in utilization of CS injections and decrease in utilization of physiotherapy among physicians treating LE. Among plastic and orthopaedic surgeons, CS injection use for tennis elbow increased from 550 per 1,000 treatments in 2009 to 663 per 1,000 treatments in 2015, whereas use of PT decreased from 406 per 1,000 treatments to 300 per 1,000 treatments over the same span.^31^ These findings, in conjunction with our own, indicate two important changes in physician practices in treatment of LE.

First, although the aforementioned study noted a relative increase in CS utilization compared with other treatment modalities, it did not address the overall utilization among all patients with LE, including those receiving no treatment. Our study demonstrated an overall decrease in CS usage, along with no notable changes of utilization of the other treatment modalities of PT, bracing treatment, and surgery. Therefore, these findings suggest a growing number of LE cases that are treated with a wait-and-see approach, which has been reported to be equally as effective in long-term treatment of tennis elbow as CS injections and physiotherapy.^20^

Second, our study highlights a population of providers who may be reluctant to change their practice in the face of growing evidence against the use of CSs for tennis elbow. This is apparent in the aforementioned increase in usage of CS injections relative to other treatments and our own finding of an increasing number of injections received per patient, among patients receiving injections. Numerous examples of the slow adoption of new evidence litter the medical landscape with many changes sometimes taking years, for example, the reported decrease in rates of knee arthroscopy for patients with osteoarthritis after an impactful publication.^35^ Further promulgation and acceptance of decreasing CS usage among providers can benefit from evidence-based guidelines published by professional organizations because such guidelines have been shown to result in notable changes in practice.^36^

Clearly, CS injection remains a popular treatment modality for LE. One aspect we did not evaluate that may further cloud the picture is treatment preferences based on specialty and subspecialty. Surveys of orthopaedic surgeons at two major international conferences in 2011 showed that 38% of orthopaedic surgeons still recommended CSs as treatment of LE, making it the most common treatment modality, tied with NSAIDs. This survey also reported CSs as a recommended treatment by 30% of hand surgeons and second only to NSAIDs (48%).^37^ Another confounding aspect is patient preference because those with past injections may request repeat injections from their physician, desiring a “quick fix” to their elbow pain, whether true or placebo.

This study used a national database, allowing access to a large sample population. However, the use of a database also comes with limitations. We were unable to gain insight into the severity of LE for ratings of pain, duration of symptoms, and level of impairment of daily activities. In addition, other epidemiological factors such as height, weight, activity level, and occupation were not able to be analyzed in this study. These factors may ultimately play a role in physician decision making. Although the database provided an overview of multiple care centers, it also relies on the accuracy of the provider in coding various practices. Furthermore, although it includes patients from all payer types such as commercial insurance, self-pay, Medicare, and Medicaid, the database remains subject to selection bias and cannot track patients who enter or exit the selection of providers available for review. Moreover, the earliest patient records provided in the database were from 2010, hindering investigation of the cohort's utilization of treatment modalities before the publication of the impactful studies on CS use.

## Conclusions

Our analysis shows that the usage of CS injections for treatment of LE has decreased from 2010 to 2017. However, there was an increase in the average number of injections among patients with LE receiving injections. These findings were consistent across age and sex and demonstrate that although there has been a decrease in overall usage of corticosteroids because of the growing quantity of impactful literature, many providers' practice has remained unaffected.

## Supplementary Material

SUPPLEMENTARY MATERIAL
