# Supplementary material for: Trends in Corticosteroid Injections for Treatment of Lateral Epicondylitis: An Analysis of 80,169 Patients
Source: J Am Acad Orthop Surg Glob Res Rev. 2021 Sep 10;5(9):e21.00186. doi: 10.5435/JAAOSGlobal-D-21-00186 (PMC8437211; doi:10.5435/JAAOSGlobal-D-21-00186)
Supplement: SUPPLEMENTARY MATERIAL [file jagrr-5-e21.00186-s001.docx]

**Table 2**. Diagnosis Trends for Lateral Epicondylitis 2010 – 2017 Among 116,454 Total Patients

|  | **2010** | **2011** | **2012** | **2013** | **2014** | **2015** | **2016** | **2017** | **p** |
| --- | --- | --- | --- | --- | --- | --- | --- | --- | --- |
| **Overall** | | | | | | | | | |
| **New Diagnoses** | 16,046 | 15,138 | 14,674 | 15,455 | 15,861 | 14,640 | 12,929 | 11,711 | **0.018** |
| **Total Patients** | 9,873,293 | 10,547,678 | 10,827,706 | 11,158,039 | 11,304,111 | 11,299,825 | 11,179,859 | 11,010,096 |  |
| **Incidence per 10,000** | 16.25 | 14.35 | 13.55 | 13.85 | 14.03 | 12.96 | 11.56 | 10.64 | <**0.01** |
| **Gender** | | | | | | | | | |
| **Male**  **(%)** | 7,188 (44.8%) | 6,723 (44.4%) | 6,616 (45.1%) | 7,018 (45.4%) | 7,049 (44.4%) | 6,420 (43.9%) | 5,518 (42.7%) | 5,119 (43.7%) | 0.052 |
| **Female (%)** | 8,858 (55.2%) | 8,415 (55.6%) | 8,058 (54.9%) | 8,437 (54.6%) | 8,812 (55.6%) | 8,220 (56.2%) | 7,411 (57.3%) | 6,592 (56.3%) |  |
| **Age** | | | | | | | | | |
| **< 25 (%)** | 257 (1.6%) | 284 (1.9%) | 324 (2.2%) | 354 (2.3%) | 350 (2.2%) | 290 (2.0%) | 239 (1.9%) | 229 (2.0%) | 0.635 |
| **25-39 (%)** | 1,916 (11.9%) | 1,894 (12.5%) | 1,854 (12.6%) | 1,886 (12.2%) | 1,930 (12.2%) | 1,756 (12.0%) | 1,624 (12.6%) | 1,415 (12.1%) | 0.856 |
| **40-64 (%)** | 12,335 (76.9%) | 11,431 (75.5%) | 10,789 (73.5%) | 11,447 (74.1%) | 11,720 (73.9%) | 10,819 (73.9%) | 9.499 (73.5%) | 8,519 (72.7%) | <**0.01** |
| **64 > (%)** | 1,538 (9.6%) | 1,529 (10.1%) | 1,707 (11.6%) | 1,768 (11.4%) | 1,861 (11.7%) | 1,775 (12.1%) | 1,567 (12.1%) | 1,548 (13.2%) | <**0.01** |

**Table 3**. Trends in Corticosteroid Injection Utilization for Lateral Epicondylitis Among 80,169 Active Patients

|  | **2010** | **2011** | **2012** | **2013** | **2014** | **2015** | **2016** | **2017** | **p** |
| --- | --- | --- | --- | --- | --- | --- | --- | --- | --- |
| **Patients Receiving Injection** | 1898 | 2128 | 2143 | 2237 | 2422 | 2202 | 1908 | 1538 | 0.338 |
| **Total Diagnosed Patients** | 8134 | 9717 | 10054 | 10772 | 11766 | 11320 | 10227 | 8179 | 0.691 |
| **%** | 23.3% | 21.9% | 21.3% | 20.8% | 20.6% | 19.4% | 18.7% | 18.8% | **<0.001** |
| **Gender** | | | | | | | | | |
| **Male**  **(%)** | 831 (43.8) | 864 (40.6) | 821 (43.0) | 1014 (45.3) | 1056 (43.6) | 974 (44.2) | 813 (42.6) | 695 (45.2) | 0.279 |
| **Female (%)** | 1067 (56.2) | 1264 (59.4) | 1222 (57.0) | 1223 (54.7) | 1366 (56.4) | 1228 (55.8) | 1095 (57.4) | 842 (54.8) |  |
| **Age** | | | | | | | | | |
| **< 40 (%)** | 159 (8.4) | 195 (9.2) | 182 (8.5) | 200 (8.9) | 222 (9.2) | 179 (8.1) | 162 (8.5) | 108 (7.0) | 0.180 |
| **40-64 (%)** | 1487 (78.4) | 1672 (78.6) | 1664 (77.7) | 1730 (77.3) | 1895 (78.2) | 1728 (78.5) | 1480 (77.6) | 1212 (78.8) | 0.233 |
| **64 > (%)** | 252 (13.2) | 261 (12.2) | 297 (13.8) | 307 (13.8) | 305 (12.6) | 295 (13.4) | 266 (13.9) | 218 (14.2) | 0.175 |
| **Injections per patient** | 1.33 | 1.56 | 1.58 | 1.68 | 1.61 | 1.75 | 1.69 | 1.83 | **0.0013** |

**Table 4**. Trends in PT Utilization for Lateral Epicondylitis Among 80,169 Active Patients

|  | **2010** | **2011** | **2012** | **2013** | **2014** | **2015** | **2016** | **2017** | **p** |
| --- | --- | --- | --- | --- | --- | --- | --- | --- | --- |
| **Overall** | | | | | | | | | |
| ***n*** | 1206 | 1472 | 1521 | 1637 | 1769 | 1658 | 1589 | 1328 | 0.449 |
| **% Diagnosed** | 14.83 | 15.15 | 15.13 | 15.20 | 15.03 | 14.65 | 15.54 | 16.24 | 0.108 |
| **Gender** | | | | | | | | | |
| **Male**  **(%)** | 458 (38.0) | 538 (36.6) | 564 (37.1) | 604 (36.9) | 690 (39.0) | 643 (38.8) | 591 (37.2) | 498 (37.5) | 0.580 |
| **Female (%)** | 748 (62.0) | 934 (63.4) | 957 (62.9) | 1033 (63.1) | 1079 (61.0) | 1015 (61.2) | 998 (62.8) | 830 (62.5) |  |
| **Age** | | | | | | | | | |
| **< 40 (%)** | 113 (9.4) | 174 (11.8) | 153 (10.1) | 169 (10.3) | 193 (10.9) | 168 (10.1) | 161 (10.1) | 143 (10.8) | 0.840 |
| **40-64 (%)** | 966 (80.1) | 1180 (80.2) | 1197 (78.7) | 1296 (79.2) | 1374 (77.7) | 1301 (78.5) | 1249 (78.6) | 1003 (75.5) | **0.011** |
| **64 > (%)** | 127 (10.5) | 118 (8.0) | 171 (11.2) | 172 (10.5) | 202 (11.4) | 189 (11.4) | 179 (11.3) | 182 (13.7) | **0.035** |

**Table 5**. Trends in Bracing Utilization for Lateral Epicondylitis Among 80,169 Active Patients

|  | **2010** | **2011** | **2012** | **2013** | **2014** | **2015** | **2016** | **2017** | **p** |
| --- | --- | --- | --- | --- | --- | --- | --- | --- | --- |
| **Overall** | | | | | | | | | |
| ***n*** | 225 | 266 | 193 | 237 | 271 | 274 | 244 | 164 | 0.646 |
| **% Diagnosed** | 2.77 | 2.74 | 1.92 | 2.20 | 2.3 | 2.42 | 2.39 | 2.01 | 0.185 |
| **Gender** | | | | | | | | | |
| **Male**  **(%)** | 97 (43.1) | 86 (32.3) | 81 (42.0) | 103 (43.5) | 108 (39.9) | 115 (42.0) | 95 (38.9) | 63 (38.4) | 0.948 |
| **Female (%)** | 128 (55.9) | 180 (67.7) | 112 (58.0) | 134 (56.5) | 163 (60.1) | 159 (58.0) | 149 (61.1) | 101 (61.6) |  |
| **Age** | | | | | | | | | |
| **< 40 (%)** | 18 (8.0) | 36 (13.5) | 12 (6.2) | 26 (11.0) | 28 (10.3) | 24 (8.8) | 25 (10.3) | 16 (9.8) | 0.928 |
| **40-64 (%)** | 178 (79.1) | 209 (78.6) | 160 (82.9) | 198 (83.5) | 216 (79.7) | 221 (80.7) | 191 (78.3) | 125 (76.2) | 0.344 |
| **64 > (%)** | 29 (12.9) | 21 (7.9) | 21 (10.9) | 13 (5.5) | 27 (10.0) | 29 (10.6) | 28 (11.5) | 23 (14.0) | 0.444 |

**Table 6**. Trends in Surgery Utilization for Lateral Epicondylitis Among 80,169 Active Patients

|  | **2010** | **2011** | **2012** | **2013** | **2014** | **2015** | **2016** | **2017** | **p** |
| --- | --- | --- | --- | --- | --- | --- | --- | --- | --- |
| **Overall** | | | | | | | | | |
| ***n*** | 274 | 324 | 369 | 375 | 361 | 368 | 345 | 234 | 0.798 |
| **% Diagnosed** | 3.37 | 3.33 | 3.67 | 3.48 | 3.07 | 3.25 | 3.37 | 2.86 | 0.122 |
| **Gender** | | | | | | | | | |
| **Male**  **(%)** | 106 (38.7) | 134 (41.4) | 160 (43.4) | 166 (44.3) | 147 (40.7) | 144 (39.1) | 132 (38.3) | 89 (38.0) | 0.268 |
| **Female (%)** | 168 (61.3) | 190 (58.6) | 209 (56.6) | 209 (55.7) | 214 (59.3) | 224 (60.9) | 213 (61.7) | 145 (62.0) |  |
| **Age** | | | | | | | | | |
| **< 40 (%)** | 29 (10.6) | 38 (11.7) | 42 (11.4) | 31 (8.3) | 38 (10.5) | 49 (13.3) | 32 (9.3) | 19 (8.1) | 0.395 |
| **40-64 (%)** | 228 (83.2) | 278 (85.8) | 306 (82.9) | 329 (87.7) | 305 (84.5) | 299 (81.3) | 293 (84.9) | 203 (86.8) | 0.695 |
| **64 > (%)** | 17 (6.2) | 8 (2.5) | 21 (5.7) | 15 (4.0) | 18 (5.0) | 20 (5.4) | 20 (5.8) | 12 (5.1) | 0.591 |

Appendix

*Lateral Epicondylitis*:

ICD-9-D-72632, ICD-10-D-M7710, ICD-10-D-M7711, ICD-10-D-M7712

*Injections*:

CPT-20550, CPT-20551, CPT-20605, CPT-20610

*Corticosteroid Dosage Codes:*

CPT-J3300, CPT-J3301, CPT-J1020, CPT-J1030, CPT-J1040

*Physical Therapy Codes:*

CPT-97110, CPT-97140, CPT-97112, CPT-97530, CPT-97010, CPT-97014, CPT-G0281, CPT-G0282, CPT-G0283, CPT-97035, CPT-97161, CPT-97162, CPT-97163, CPT-97165, CPT-97166, CPT-97167, CPT-97168, CPT-97535, CPT-97016, CPT-92507, CPT-97032, CPT-97164, CPT-97012, CPT-97150, CPT-97113, CPT-97124, CPT-0101T, CPT-0102T, CPT-0512T, CPT-0513T, CPT-28890, CPT-97039, CPT-97026, CPT-97799, CPT-97901, CPT-97139, CPT-S8948

*Bracing Codes:*

CPT-L3702, CPT-L3710, CPT-L3720, CPT-L3730, CPT-L3740, CPT-L3760, CPT-L3761, CPT-L3762, CPT-97760, CPT-97762

*Surgery Codes:*

CPT-24006, CPT-24101, CPT-24102, CPT-24350, CPT-24351, CPT-24352, CPT-24353, CPT-24354, CPT-24355, CPT-24356, CPT-24357, CPT-24358, CPT-24359, CPT-CPT-29834, CPT-29835, CPT-29836, CPT-29837, CPT-29838, CPT-29999
